# Emerging roles of histone methylation in phytopathogenic fungi

**DOI:** 10.1007/s42994-025-00223-6

**Published:** 2025-07-02

**Authors:** Qi Zhang, Zeng Tao

**Affiliations:** 1 Ministry of Agriculture and Rural Affairs Key Laboratory of Molecular Biology of Crop Pathogens and Insect Pests, Institute of Biotechnology, Zhejiang University, Hangzhou, 310058 China; 2 Zhejiang Key Laboratory of Biology and Ecological Regulation of Crop Pathogens and Insects, Zhejiang Engineering Research Center for Biological Control of Crop Pathogens and Insect Pests, Zhejiang University, Hangzhou, 310058 China

**Keywords:** Fungal development, Histone methylation, H3K4 methylation, H3K27 methylation, H3K36 methylation, Phytopathogenic fungi

## Abstract

Plant-pathogenic fungi significantly affect crop yield and quality. Understanding pathogenic mechanisms and reducing yield losses from plant diseases are therefore crucial for global food security. Epigenetics has become a central focus in fungal biology research, and recent refinements in high-throughput sequencing technologies have drawn attention to the role of histone methylation in fungal pathogenicity. Due to their diversity and complexity, histone methylations play crucial roles in epigenetic and transcriptional regulation. In this review, we summarize recent progress in understanding histone methylation in plant-pathogenic fungi and examine how these modifications influence fungal pathogenicity. Ultimately, we aim to offer insight for creating fungal disease control strategies through the lens of histone methylation.

## Introduction

Filamentous fungal pathogens pose a persistent threat to global agricultural productivity, with plant-pathogenic species causing devastating yield losses and deterioration in the quality of major crops. Three particularly destructive taxa highlight this issue. *Magnaporthe oryzae* is the causal agent of rice blast disease, which destroys 10%–30% of global rice production annually (Fernandez and Orth 2018). *Botrytis cinerea*, a necrotrophic generalist, infects more than 1,400 plant species, including economically vital fruit crops, and causes billions of dollars in annual economic losses (Dwivedi et al. 2024). *Fusarium* species infect diverse monocot and dicot hosts through mycotoxin production and vascular colonization, severely compromising both crop yield and food safety (Nag et al. 2022; Zakaria 2023). Mitigating the dual challenges of crop protection and post-harvest management against these pathogens remains a paramount objective of modern plant pathology and agricultural sustainability research. To establish successful infection and colonization, phytopathogenic fungi employ sophisticated strategies that rely on spatiotemporally regulated gene expression programs (Toruno et al. 2016; Xie and Duan 2023).

Epigenetics refers to mitotically and meiotically heritable modifications that regulate gene expression through chemical alterations to DNA bases, histone proteins, and non-coding RNAs without altering DNA nucleotide sequences (Shilpa et al. 2022). Emerging evidence indicates that epigenetic modifications play critical roles in regulating both developmental programs and virulence mechanisms in fungal phytopathogens (Dubey and Jeon 2017; Gomez-Diaz et al. 2012). In eukaryotic cells, genomic DNA is condensed into a highly organized chromatin structure. The nucleosome, the fundamental unit of chromatin, comprises approximately 146 base pairs of DNA wrapped around a histone octamer containing two copies of each subunit: H2A, H2B, H3, and H4 (Peterson and Laniel 2004; Sullivan et al. 2006). Post-translational modifications on the N-terminal tails of histones, including methylation, acetylation, phosphorylation, and ubiquitination, play key roles in regulating chromatin structure, consequently influencing the accessibility of DNA to the transcriptional machinery (Lai et al. 2022).

In general, chromatin regions exhibiting higher accessibility are more transcriptionally active, whereas those with reduced accessibility tend to impose spatial constraints on the transcriptional machinery. Chromatin with relatively high accessibility (predominantly classified as euchromatin) facilitates transcriptional activation, while regions with lower accessibility (heterochromatin) establish transcriptional silencing via stable chromatin compaction (Bannister and Kouzarides 2011). Notably, accessibility gradients exist within euchromatin domains, and certain epigenetic modifications can still occur in heterochromatic regions. This plasticity of chromatin enables precise regulation of intracellular processes, phenotypic adaptation in response to environmental stimuli, and interplay between the host and pathogen (Kang et al. 2022).

Among epigenetic regulators, histone post-translational modifications exhibit unparalleled functional diversity, which is mechanistically linked to specific transcriptional outputs (Millán-Zambrano et al. 2022). For example, histone acetylation is universally associated with chromatin relaxation and transcriptional activation, whereas histone methylation exhibits context-dependent effects governed by both site specificity and the methylation state (Rymen et al. 2019). Repressive marks such as H3K9me2/3 (di- or tri-methylation on histone 3 lysine 9) and H3K27me2/3 demarcate transcriptionally silent heterochromatin, whereas activating marks including H3K4me3 and H3K36me3 characterize transcriptionally active euchromatin (Latham and Dent 2007). The dynamic nature of chromatin architecture allows histone-modifying enzymes to establish complex regulatory patterns through two principal mechanisms: (i) cooperative or antagonistic crosstalk between distinct modifications, at identical genomic loci; and (ii) combinatorial deposition of multiple modifications across different chromatin regions. These sophisticated regulatory mechanisms, which collectively constitute the “histone code” (Zhao et al. 2019), orchestrate precise spatiotemporal control of transcriptional networks, thereby modulating cellular processes and phenotypic plasticity.

In this review, we summarize recent advances in understanding epigenetic regulatory mechanisms in filamentous fungi, with a particular emphasis on phytopathogenic species. We focus on elucidating dynamic changes in histone methylation patterns that govern critical biological processes, including fungal development and virulence acquisition. Historically, foundational discoveries concerning histone-modifying enzymes have predominantly originated from model organisms such as the yeast *Saccharomyces cerevisiae* and non-pathogenic fungal systems. The rapid reproductive cycles and genetic tractability of fungal systems have accelerated mechanistic investigations, allowing the rapid validation of epigenetic regulatory paradigms. Below, we summarize recent breakthroughs in fungal epigenetics and plant pathology through cross-species comparative analyses.

## Types of histone methylation

Histone modification represents a fundamental mechanism of epigenetic regulation, influencing chromatin architecture and gene expression through post-translational covalent modifications. Histone methylation exhibits distinct functional states through mono- (me1), di- (me2), and tri-methylation (me3) at specific lysine residues, which are associated with different regulatory outputs. For instance, H3K4me3 is predominantly enriched at the promoters of active genes, facilitating transcription initiation (Bernstein et al. 2006), while H3K36me3 localizes to gene bodies, coordinating transcription elongation and co-transcriptional mRNA processing (Li et al. 2007). H4K20me plays various roles, functioning in heterochromatin compaction and euchromatic DNA repair (Beck et al. 2012). H3K27me3, catalyzed by Polycomb repressive complexes, accumulates at the promoters and bodies of development-related genes to enforce transcriptional silencing (Margueron and Reinberg 2011). H3K79me2 occupies mid-genic regions and may regulate transcriptional fidelity and DNA damage repair. By contrast, H3K9me3 demarcates heterochromatin and repetitive elements, thereby mediating chromatin stability and heritable gene repression (Bernstein et al. 2006).

**Fig. 1:**
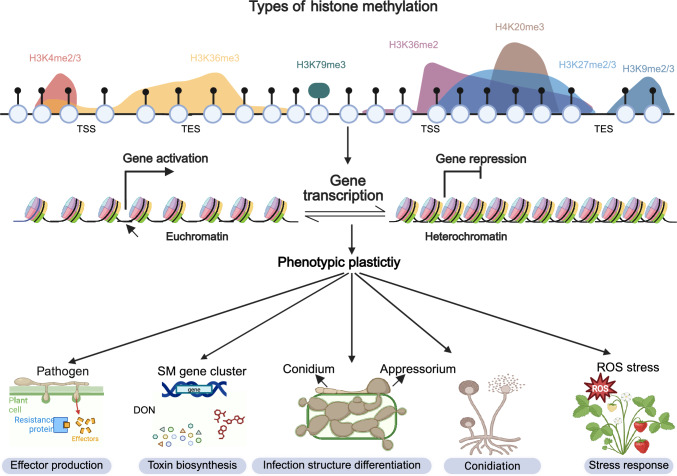
Schematic model of histone lysine methylation and its regulatory roles in filamentous fungal pathogens. The detailed regulatory mechanisms are described in the main text. Different types of histone methylation exert distinct effects on gene expression. In general, H3K4me2/3 and H3K36me3 contribute to chromatin relaxation, facilitating transcription factor binding and promoting gene activation. By contrast, H3K27me2/3, H3K36me2, H3K9me2/3, H3K79me3, and H4K20me3 lead to chromatin compaction, restricting transcription factor accessibility and repressing gene expression. Gene transcription mediated by histone modification ultimately influences various biological processes, including fungal growth and development, pathogenicity, the secretion of effector proteins, secondary metabolite biosynthesis, and stress responses. SM, secondary metabolite; ROS, reactive oxygen species; DON, deoxynivalenol

Emerging evidence highlights the complicated crosstalk among these modifications. While H3K4me3 and H3K36me3 demonstrate spatial co-localization across actively transcribed genes, H3K27me3 and H3K9me3 exhibit mutually exclusive genomic targeting despite their shared repressive functions, reflecting their engagement with distinct chromatin domains and biological processes (Ferrari et al. 2014; van Rossum et al. 2012). Such interplay underscores the combinatorial nature of the “histone code”, which dynamically orchestrates cellular differentiation, development, and disease states. The proper maintenance and crosstalk of histone modifications are critically important in fungi, influencing their growth and development, pathogenic capacity, secondary metabolism, and responses to abiotic stress (Fig. 1, Table 1).

**Table 1:** Pathogenic fungal species included in this review and the impact of histone methylation on their growth and pathogenicity

Fungal Species	Enzyme	Modification	Growth	Virulence	Reference
*Neurospora crassa*	Set7	H3K27me2/3	–	ND	Jamieson et al. (2013)
	DIM5	H3K9me3	–	ND	Gessaman and Selker (2017)
	Set2/Ash1	H3K36me	–	ND	Bicocca et al. (2018)
*Fusarium graminearum*	KMT6	H3K27me3	–	–	Connolly et al. (2013)
	Set1	H3K4me	–	–	Liu et al. (2015)
	KMT5	H4K20me	–	–	Bachleitner et al. (2021)
*Magnaporthe oryzae*	KMT6	H3K27me3	ND	–	Wu et al. (2022)
	Set1	H3KK4me3	–	–	Pham et al. (2015)
	Set2/Ash1	H3K36me2/3	–	–	Xu et al. (2024a)
	KMT5	H4K20me	ND	ND	Pham et al. (2015)
*Dothistroma septosporum*	KMT6	H3K27me3	–	–	Chettri et al. (2018)
*Epichloë festucae*	EzhB	H3K27me3	–	–	Chujo and Scott (2014)
	ClrD	H3K9me3	–	–	Chujo and Scott (2014)
*Ustilaginoidea virens*	KMT6	H3K27me3	–	–	Meng et al. (2021)
*Leptosphaeria maculans*	DIM5	H3K9me3	–	–	Soyer et al. (2014)
*Beauveria bassiana*	DIM5	H3K9me3	–	–	Atanasoff–Kardjalieff et al. (2021)
	Set1	H3K4me	–	–	Ren et al. (2021b)
	Set2/Ash1	H3K36me2/3	–	–	Ren et al. (2021c)
*Colletotrichum higginsianum*	CclA	H3K4me	–	–	Dallery et al. (2019)
*Fusarium fujikuroi*	Set1	H3K4me	–	–	Janevska et al. (2018b)
	Set2/Ash1	H3K36me2/3	–	–	Janevska et al. (2018a)
*Fusarium verticillioides*	Set2	H3K36me3	–	–	Gu et al. (2017)
*Aspergillus flavus*	SetB/AshA	H3K36me2/3	–	–	Zhuang et al. (2022)
	Dot1	H3K79me3	–	–	Liang et al. (2017)
	Set9	H4K20me			Hao et al. (2023)
*Penicillium oxalicum*	Dot1	H3K79me3	–	–	Li et al. (2019)
*Schizosaccharomyces pombe*	KMT5	H4K20me	ND	ND	Wang and Jia (2009)
*Zymoseptoria tritici*	KMT5	H4K20me	–	ND	Moller et al. (2023)

−, Negative regulation; ND, not detected/not different

### H3K27 methylation: a facultative heterochromatin mark associated with transcriptional repression

In eukaryotes, heterochromatin exists in two distinct forms: facultative heterochromatin, a form primarily characterized by H3K27 methylation; and constitutive heterochromatin, which is marked by H3K9 methylation (Bell et al. 2023). Genes within facultative heterochromatin display transcriptional plasticity, enabling dynamic changes in expression in response to environmental stimuli, thus facilitating organismal adaptation. Constitutive heterochromatin predominantly occupies evolutionarily conserved genome regions, including centromeres and telomeres, helping to maintain genome stability (Janssen et al. 2018). This functional specialization allows the two types of heterochromatin to play crucial roles in regulating gene expression and maintaining genome stability, which are essential for organism survival in changing environments (Bell et al. 2023). During host invasion, pathogenic fungi regulate H3K27me modifications to control pathogenicity. Reduced H3K27me marks at virulence-associated loci activate the transcription of genes that are typically silenced, driving the production of effector proteins and enzymes for host colonization. Loss of Polycomb Repressive Complex 2 (PRC2) and H3K27me erasure leads to changes in gene expression and pathogenicity, with both direct and indirect effects. This strategy is conserved across fungal pathogens, highlighting its evolutionary role in host–pathogen co-evolution (Zhang et al. 2021).

In multicellular eukaryotes, H3K27me is deposited by Polycomb group (PcG) proteins, which assemble into Polycomb Repressive Complex 1 (PRC1) and PRC2 and alter chromatin accessibility, thereby suppressing gene expression (Wiles and Selker 2017). PRC1 is an E3 ubiquitin ligase complex that catalyzes the ubiquitination of lysine residues on histone H2A and mediates chromatin compaction through its catalytic H2AK119 ubiquitination activity and steric chromatin crosslinking (Endoh et al. 2008). Structural studies have revealed that dimer formation of the PRC1 subunits Ring1B/YY1 induces phase separation, increasing nucleosome density at target loci through multi-valent interactions (Isono et al. 2013). This condensed architecture physically obstructs transcription factor binding and impedes RNA polymerase II (RNAPII) processivity (Kundu et al. 2017). Cryo-ET analysis demonstrated that chromatin fiber diameters are smaller in PRC1-enriched regions than in active regions, correlating with H2AK119ub deposition (Boyle et al. 2020). PRC1 also recruits histone variants and HDACs to stabilize repressive histone modifications, creating self-reinforcing silencing loops (Schjoldager et al. 2020).

In animals, PRC2, a histone methyltransferase complex (Laugesen et al. 2019), catalyzes H3K27me1, 2, and 3: H3K27me2 and 3 repress gene expression while H3K27me1 promotes gene expression (Jacob and Michaels 2009; Shen et al. 2008). In plants, PRC2 only mediates H3K27me2/3 deposition to repress gene expression, whereas H3K27me1 is specifically catalyzed by Arabidopsis (*Arabidopsis thaliana*) Trithorax-Related5/6 (ATXR5/6) to repress gene expression (Jacob et al. 2009). Although most fungi contain the core subunits of PRC2, no fungal genome examined to date contains the key components of canonical PRC1 and, currently, there are no reports on the ubiquitination of H2A in fungi. Only the functions of H3K27me2/3 in fungi have been reported, with little to no information available on H3K27me1 in fungi. Thus, the mechanisms by which H3K27me regulates transcription in fungi is not completely characterized.

In the red bread mold *Neurospora crassa*, PRC2 comprises the methyltransferase Enhancer of Zeste Homolog (EZH), which contains a Set domain and belongs to the Lysine Methyltransferase 6 (KMT6) family, along with the core subunits Embryonic Ectoderm Development (Eed) and Suppressor of Zeste 12 (Suz12) and the additional subunit Npf (P55). KMT6, Eed, and Suz12 are essential for H3K27 methylation, as their loss leads to a complete loss of H3K27me3 (Jamieson et al. 2013). Npf is important, but not indispensable, as its deletion only reduces and redistributes H3K27 methylation across the chromatin (Jamieson et al. 2013). However, the loss of these core subunits or additional subunit leads to dramatic changes in transcription.

H3K27me serves as a repressive mark, repressing gene expression in fungi (Moser Tralamazza et al. 2022). However, unlike H3K9me, which is typically localized to constitutive heterochromatin, H3K27me is primarily found in facultative heterochromatin within gene bodies across the genome. Genes marked by facultative heterochromatin can undergo transcriptional changes in response to changes in environmental conditions (Trojer and Reinberg 2007). The proper expression of genes marked by H3K27me3 is crucial for fungal growth, development, and pathogenicity. In fungi, secondary metabolite gene clusters are frequently marked by H3K27me3 to regulate the expression of genes involved in secondary metabolism (Yu et al. 2023). Despite the rapid increase in experimental evidence for H3K27 methylation in fungi, functional analysis of H3K27me remains constrained by unresolved technical barriers to mapping its dynamic patterns in repetitive genomes and the absence of genetic tools targeting Polycomb/Trithorax homologs in non-model pathogens (Ridenour et al. 2020).

Loss of H3K27me can affect fungal growth. For instance, deletion of core PRC2 subunits such as KMT6, Eed, or Suz12 in *Fusarium graminearum* causes severe defects in hyphal growth and development (Connolly et al. 2013). These phenotypic defects might be attributed to the inappropriate expression of genes involved in various pathways related to signal transduction and asexual growth, which are marked by H3K27me3. The loss of H3K27me also has a notable impact on fungal pathogenicity (Ridenour et al. 2020). In *M. oryzae*, the depletion of H3K27me3 causes a severe reduction in spore production, impaired appressorium formation, disrupted effector protein expression, and significantly decreased pathogenicity on rice (*Oryza sativa*) (Connolly et al. 2013; Wu et al. 2022).

The accessory PRC2 subunit P55 plays crucial roles in maintaining the proper genomic distribution of H3K27me3 and in transcriptional repression in *M. oryzae*. Upon deletion of P55, genes previously bound by H3K27me3 are de-repressed, with differences in gene expression highly overlapping with those observed in a KMT6 mutant. Moreover, P55 is essential for the proper genomic occupancy of H3K27me3 (Lin et al. 2022). In *Dothistroma septosporum*, loss of H3K27me3 activates the expression of genes required for the production of the secondary metabolite dothistromin, which are transcriptionally silenced during growth (Chettri et al. 2018). This de-repression has a series of effects on the growth, development, and pathogenicity of this fungus (Chettri et al. 2018). Additionally, removal of H3K27me3 has a profound impact on the colonization ability of the symbiotic fungus *Epichloë festucae* (Chujo and Scott 2014). A similar role for Kmt6-mediated H3K27 methylation in regulating fungal virulence has been described in both *Ustilaginoidea virens* and *F. graminearum* (Connolly et al. 2013; Meng et al. 2021).

Given the absence of PRC1 in fungi, exploring how H3K27me3-mediated facultative heterochromatin is established and how stable transcriptional silencing is achieved will be valuable. The diversity and complexity of H3K27me3-regulatory mechanisms is an important area of focus in the epigenetics community (Wu and Fan 2025). In *F. graminearum*, the identification of a conserved BAH–PHD protein 1 (BP1) as a reader protein elegantly demonstrated how H3K27me is recognized (Tang et al. 2021)*.* BP1 interacts with the core PRC2 subunit Suz12 and directly binds to methylated H3K27. BP1 exhibits DNA binding activity through its PHD domain, contributing to the maintenance of transcriptional repression in H3K27me3-marked genes (Tang et al. 2021). The intrinsically disordered region 2 (IDR2) of BP1 was recently found to mediate the liquid–liquid phase separation of this protein, which is essential for the interaction between BP1 and the PRC2 subunit Suz12, as well as for the recognition and binding of H3K27me3 (Tang et al. 2024). In addition, a study of three-dimensional genome organization and histone modifications in *F. graminearum* revealed that genomic regions occupied by Polycomb proteins frequently coincide with regions enriched in H3K27me3, thereby facilitating unique transcriptional interactions within the genome (Shao et al. 2024). From an evolutionary perspective, H3K27me3 predominantly marks less conserved genomic regions and is enriched at genomic rearrangement breakpoints, suggesting a potential role in maintaining genome stability (Shao et al. 2024).

### H3K9 methylation: a repressive histone mark primarily associated with constitutive heterochromatin and gene silencing

**Fig. 2:**
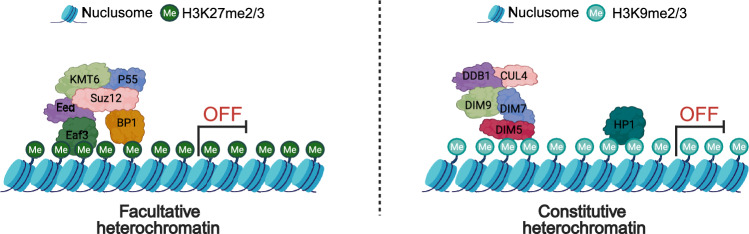
Model of the catalytic mechanisms regulating H3K27 and H3K9 methylation. H3K27me is mediated by the Polycomb Repressive Complex 2 (PRC2), composed of the core subunits Kmt6, Eed, and Suz12. The accessory protein P55 interacts with both Eed and Suz12, contributing to the structural integrity and chromatin recruitment of PRC2. The H3K36me reader Eaf3 binds to Eed and facilitates PRC2 localization to specific genomic regions, thereby promoting H3K27 methylation. BP1, a reader of H3K27me, interacts with Suz12 and reinforces PRC2 activity through a positive feedback mechanism, enhancing the spread and maintenance of H3K27me marks across chromatin. H3K9me is catalyzed by the histone methyltransferase DIM-5. Proper targeting of DIM-5 to chromatin depends on DIM-7 and a Cullin4-based E3 ubiquitin ligase complex composed of DIM-9, DDB1, and CUL4, which together facilitate recruitment and stabilization of DIM-5 at heterochromatic regions. The resulting H3K9me2/3 marks are recognized by the heterochromatin protein HP1, which promotes the establishment of transcriptionally repressive heterochromatin and reinforces DIM-5–mediated silencing

H3K9me is catalyzed by Lysine Methyltransferase 1 (KMT1) proteins, which constitute the largest family of histone lysine methyltransferases (Freitag 2017) that includes Cryptic loci regulator 4 (Clr4) in *Schizosaccharomyces pombe* and Defective in Methylation 5 (DIM5) in *N. crassa*(Fig. 2). In fungi, the loss of H3K9me leads to genome-wide transcriptional changes and significant defects in growth, development, and pathogenicity (Ren et al. 2021a). In *E. festucae*, H3K9me3 regulates the biosynthesis of lolitrem and ergot alkaloids, ensuring that these bio-protective metabolites are produced exclusively during the symbiotic association between *E. festucae* and perennial ryegrass (*Lolium perenne*) (Chujo and Scott 2014). However, upon the loss of H3K9me3, expression of lolitrem and ergot alkaloid biosynthetic genes is de-repressed, leading to their expression at other developmental stages (Chujo and Scott 2014).

In *Leptosphaeria maculans*, RNAi-mediated silencing of *LmDIM5* leads to a complete absence of chromatin condensation (Soyer et al. 2014). Furthermore, numerous genes are significantly upregulated in *Heterochromatin Protein 1* (*LmHP1*)-silenced or *LmDIM5*-silenced strains under axenic growth conditions, including genes encoding small secreted proteins (Soyer et al. 2014). In *Beauveria bassiana*, deletion of the *DIM5* gene leads to the complete loss of H3K9me3 and the differential expression of thousands of genes (Ren et al. 2021a). Moreover, the mutant displays severe defects in multiple cellular processes related to fungal virulence and lifecycle, including significantly reduced hyphal growth, blocked conidial formation, impaired in vivo proliferation, altered carbohydrate epitopes, a disrupted cell cycle, the diminished biosynthesis and secretion of cuticle-degrading enzymes, and increased sensitivity to various stresses (Ren et al. 2021a). Furthermore, the mutant has completely lost its ability to infect insects through normal cuticle penetration, and its virulence is substantially reduced even when infection is initiated via direct hemocoel injection (Ren et al. 2021a).

Although H3K27 and H3K9 methylation represent distinct types of heterochromatin marks, they exhibit intricate interconnections. In *N. crassa*, deletion of the H3K9 methyltransferase leads to the loss of H3K27me3 in many facultative heterochromatic regions, whereas H3K27me3 appears in constitutive heterochromatic regions that were originally marked by H3K9me3 (Jamieson et al. 2016). Additionally, deletion of the H3K9me3 reader gene *HP1* leads to the redistribution of H3K27me3 methylation, resulting in less H3K27me3 in facultative heterochromatic regions and the appearance of H3K27me2 in constitutive heterochromatic regions (Jamieson et al. 2016). Notably, while both H3K27me3 and H3K9me3 contribute to heterochromatin formation, H3K27me3 cannot functionally compensate for the loss of H3K9me3 (Jamieson et al. 2016).

### H3K4 methylation: an active histone mark enriched at gene promoters (H3K4me2/3) and enhancers (H3K4me1)

**Fig. 3:**
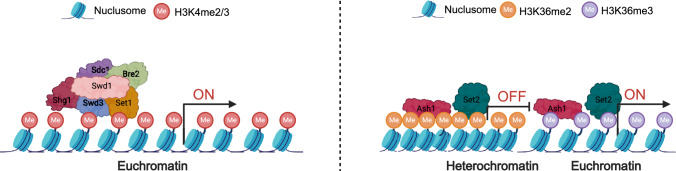
Schematic model of the catalytic mechanisms of H3K4me3 and H3K36me. H3K4me3 is catalyzed by the Set1/COMPASS complex, with Set1 serving as the core methyltransferase. Swd1 and Swd3 maintain the structural stability of the complex, while Sdc1 and Bre2 enhance catalytic activity, and Shg1 assists in complex assembly. H3K4me3 primarily marks active gene promoters and promotes gene expression. H3K36 methylation is catalyzed by Ash1 and Set2. Among the methylation states, H3K36me2 generally represses gene expression, whereas H3K36me3 promotes gene expression, with both modifications playing distinct roles in transcriptional regulation

H3K4me is catalyzed by KMT2 methyltransferases such as SET Domain Containing 1 (Set1), which is a representative KMT2 protein. Set1 is a core component of the Complex of Proteins Associated with Set1 (COMPASS), which was initially discovered in yeast and is highly conserved in fungi (Shilatifard 2008). The COMPASS consists of Set1 and the subunits Swd3, Bre2, and Swd1, which work together to maintain H3K4 methylation (Ruthenburg et al. 2007). In this process, Set1 functions as the core methyltransferase, while Swd1 and Swd2 contribute to the structural stability of the complex. Sdc1 and Bre2 enhance the catalytic activity, and Shg1 facilitates the proper assembly of the COMPASS complex (Fig. 3). In addition to H3K4 methyltransferases, fungi also possess H3K4 demethylases; Lysine Demethylase 1 (KDM1) and KDM5 (Lai et al. 2020; Shilatifard 2008) have been shown to exhibit H3K4 demethylase activity. H3K4me2 and H3K4me3 are tightly linked to transcription initiation and elongation, whereas H3K4me3 is a canonical marker of active promoters, where it recruits chromatin readers to facilitate RNAPII loading and transcription initiation (Bernstein et al. 2006). While H3K4me2 overlaps with H3K4me3 at gene promoters, it also extends into gene bodies, potentially stabilizing transcription elongation through interactions with elongation complexes. Both modifications are dynamically deposited by Set1/COMPASS complexes and serve as epigenetic “memory” for recurrently activated genes (Benayoun et al. 2014). H3K4me1 is a dynamic epigenetic mark associated with enhancers and poised regulatory elements. Whereas H3K4me2/3 is linked to active promoters, H3K4me1 primes chromatin for future activation by marking loci awaiting transcriptional stimuli. H3K4me1 facilitates enhancer-promoter looping and recruits chromatin remodelers to maintain developmental gene programs or stress-responsive states (Whyte et al. 2013). Cooperative binding with pioneer transcription factors further enables lineage-specific gene activation (Calo and Wysocka 2013; Heintzman et al. 2009).

H3K4me plays critical roles in the host infection process of many pathogenic fungi. In *M. oryzae*, H3K4me is mediated by MoSet1 (Pham et al. 2015); loss of *MoSet1* abolishes H3K4 methylation, resulting in significant transcriptional alterations and lower virulence (Zhou et al. 2021). In *B. cinerea*, the H3K4 demethylase JARID1/KDM5 plays crucial roles in fungal development and pathogenicity. Deletion of *JARID1* affects conidiation and appressorium formation, leading to impaired infection cushion formation and virulence (Hou et al. 2020). In *Colletotrichum higginsianum*, deletion of *CclA*, encoding a component of the COMPASS complex, leads to lower H3K4me, resulting in less mycelial growth, conidiation, spore germination, and virulence (Dallery et al. 2019). However, a reduction in H3K4me leads to the overproduction of three distinct families of metabolic compounds that influence the ability of pathogenic bacteria to infect plants. These include five previously uncharacterized compounds in the colletotochlorin and higginsianin families whose roles in fungal growth and pathogenicity have been confirmed (Dallery et al. 2019).

In *Fusarium fujikuroi*, Set1 is responsible for all detectable H3K4me2/3, while KDM5 removes H3K4me3 (Janevska et al. 2018b). Deletion of *Set1* and *KDM5* results in enhanced transcription across the *F. fujikuroi* genome, substantially affecting the expression of key genes involved in secondary metabolism (Janevska et al. 2018b). In *F. graminearum*, H3K4 methylation mediated by FgSet1 activates the transcription of genes encoding two toxins that are closely associated with its pathogenicity, thereby influencing virulence (Liu et al. 2015). In *B. bassiana*, deletion of *Set1* leads to the complete loss of H3K4 methylation, resulting in dysregulated gene expression (Ren et al. 2021b); this significantly impairs pathogenicity, conidial hydrophobicity, and adhesion to insect exoskeletons, thereby causing severe defects in growth and conidiation (Ren et al. 2021b).

### H3K36 methylation: distinct forms of H3K36me2 and H3K36me3 with different transcriptional activities

H3K36me is a crucial histone modification closely associated with gene transcription, chromatin structure, and genome stability. H3K36 can undergo progressive methylation to form mono (H3K36me1), di (H3K36me2), and tri-methylated (H3K36me3) forms via specific histone methyltransferases; these modifications have distinct functions (Ding et al. 2022). H3K36me3 is typically enriched within the coding regions of transcriptionally active genes and is associated with RNAPII elongation (Kizer et al. 2005). H3K36me3 facilitates the recruitment of chromatin remodeling factors and histone deacetylase complexes, thereby suppressing aberrant transcription initiation and ensuring transcriptional fidelity (Lam et al. 2022). Furthermore, H3K36me plays a critical role in DNA damage repair by interacting with specific repair factors to promote homologous recombination repair (Jha and Strahl 2014).

H3K36me plays critical roles in fungal growth, development, and pathogenicity. In *N. crassa*, the H3K36 methyltransferase Set2 is closely associated with RNAPII (Bicocca et al. 2018). However, *N. crassa* also contains another H3K36 methyltransferase, Absent, Small, or Homeotic Discs 1 (Ash1) (Bicocca et al. 2018). Although both enzymes mediate the deposition of H3K36me2 and H3K36me3, their relative contributions and functional roles are strikingly different (Bicocca et al. 2018). In terms of catalytic proportions, Set2 accounts for approximately 90% of the deposition of both H3K36me2 and H3K36me3, whereas Ash1 accounts for only ~ 10% of these activities. Furthermore, Ash1-catalyzed H3K36me2 primarily targets inactive genes and plays a critical role in repressing gene expression, whereas Set2-catalyzed H3K36me3 predominantly marks active genes and promotes their transcription (Bicocca et al. 2018). Notably, when Ash1 is deleted, the expression of genes co-occupied by both Ash1 and Set2 becomes significantly higher, whereas the expression of genes exclusively associated with Set2 remains largely similar (Bicocca et al. 2018). Furthermore, Ash1 plays dual roles in modulating the accumulation of H3K27me2/3, with both positive and negative effects (Bicocca et al. 2018). Although the roles of H3K36me2 and H3K36me3 have been extensively studied, research on H3K36me1 remains relatively limited, and its biological functions and regulatory mechanisms warrant further investigation. In *Fusarium verticillioides*, deletion of *SET2* results in significant defects in vegetative growth, less fumonisin B1 biosynthesis, abnormal pigment production, and impaired fungal virulence (Gu et al. 2017).

In *F. fujikuroi*, H3K36me is also catalyzed by Set2 and Ash1, which target different genomic regions (Janevska et al. 2018a). Set2 primarily mediates H3K36me in euchromatic regions of the genome, while Ash1 is mainly responsible for H3K36 methylation at sub-telomeric regions of chromosomes (Janevska et al. 2018a). The deletion of *Ash1* results in significantly higher H3K27me3 levels (Janevska et al. 2018a). Phenotypic analysis has demonstrated that H3K36 methylation is essential for vegetative growth, sporulation, secondary metabolite biosynthesis, and pathogenicity in *F. fujikuroi* (Janevska et al. 2018a).

In *B. bassiana*, H3K36me is catalyzed by Set2 and Ash1. Set2 primarily catalyzes the tri-methylation of H3K36 in active gene regions. By contrast, Ash1 methylates H3K36 in different chromosomal regions, especially in telomeric and heterochromatic regions, thereby maintaining chromatin structure and gene silencing (Ren et al. 2021c). The loss of these two methyltransferases leads to significantly lower pathogenicity, particularly affecting processes such as immune evasion, virulence, and spore formation in the insect host (Ren et al. 2021c).

In *Aspergillus flavus*, H3K36me is primarily mediated by SetB and AshA: AshA mainly regulates H3K36me2 modification, while SetB is primarily responsible for H3K36me3 modification. Both enzymes play crucial roles in morphogenesis and mycotoxin biosynthesis as well as the pathogenicity of the fungus toward both crops and animals (Zhuang et al. 2022). In *M. oryzae*, H3K36me2/3 is catalyzed by Set2 and Ash1: H3K36me2 catalyzed by Ash1 primarily represses gene expression, whereas H3K36me3 catalyzed by Set2 mainly activates gene expression (Xu et al. 2024a). H3K36me2 marks catalyzed by Ash1 are significantly co-localized with H3K27me3 marks in the genome, and the deletion of the H3K27me3 methyltransferase KMT6 leads to lower levels of H3K36me2 (Xu et al. 2024a).

Recent studies have revealed that in *M. oryzae*, the H3K36me reader Esa1-Associated Factor 3 (Eaf3) interacts with the H3K36 methyltransferase Ash1, the core PRC2 subunit Eed, and the histone deacetylation co-suppressor Switch-Independent 3 (Sin3), thereby regulating nucleosome density in occupied regions of the genome to coordinate the repression of gene expression (Xu et al. 2024b). Experimental evidence indicates that Eaf3 plays a critical role in maintaining the functional integrity of Ash1, Eed, and Sin3 (Xu et al. 2024b). Moreover, Eaf3-occupied regions are associated with increased nucleosome occupancy, contributing to transcriptional silencing in *M. oryzae*. Therefore, Eaf3 acts as a repressive H3K36me2 reader and plays vital roles in Polycomb gene silencing and the formation of facultative heterochromatin in fungi (Xu et al. 2024b).

### H3K79 methylation: a key active mark linked to transcription, genomic stability, and cell cycle progression

H3K79me is catalyzed by Disruptor of Telomeric Silencing 1 (Dot1), which belongs to the Lysine Methyltransferase 4 (KMT4) family and was first identified in *S. cerevisiae* (Singer et al. 1998). Unlike other methyltransferases, Dot1 lacks a SET domain, and its catalytic site is not located on the histone tail but rather on the globular domain of histone H3. In *S. cerevisiae*, Dot1 prevents the nonspecific binding of Sir proteins to DNA by catalyzing H3K79 methylation, thereby ensuring proper gene expression (Vlaming and van Leeuwen 2016).

Studies on H3K79me in filamentous fungi are limited, and its precise functions remain unclear (Kitada et al. 2012; Vlaming and van Leeuwen 2016). In *Penicillium oxalicum*, PoDot1 modulates the transcription of key regulatory factors involved in asexual development, thereby affecting conidiation. PoDot1 also regulates the transcription of five septin-encoding genes, contributing to normal hyphal septum and branch formation (Li et al. 2019). Additionally, PoDot1 regulates the expression of extracellular glycoside hydrolase genes, and its deletion significantly impairs growth and development (Li et al. 2019). In *A. flavus*, deletion of *Dot1* leads to significantly lower conidiation and greater sclerotium formation, as well as altered tolerance to various stresses and aflatoxin production (Liang et al. 2017).

### H4K20 methylation: a repressive mark linked to heterochromatin

H4K20me is catalyzed by members of the KMT5 family; its catalytic mechanisms vary among species. In mammals, H4K20me1 is catalyzed by KMT5A, whereas H4K20me2/3 is catalyzed by KMT5B/C (KMT5B and KMT5C) (Hyun et al. 2017). In fungi, the single KMT5 homolog Set9 mediates H4K20me1/2/3, which are generally considered to be associated with gene silencing (Hyun et al. 2017). In *S. pombe*, Set9-catalyzed H4K20me2 plays an important role in the DNA damage response. Upon DNA damage, Checkpoint Rad9 Binding 2 (Crb2) protein binds to the exposed H4K20me2 and phosphorylated sites on H2A, thereby triggering the initiation of DNA repair (Wang and Jia 2009). In *M. oryzae*, KMT5 catalyzes H4K20me3; its deletion leads to slightly inhibited fungal growth but does not affect spore germination, conidiation, appressorium formation, or pathogenicity (Pham et al. 2015). However, the level of H4K20me3 modification is also lower in *M. oryzae* when Set1 is deleted (Pham et al. 2015).

In *F. graminearum* and *F. fujikuroi*, KMT5 mediates H4K20me1/2/3. Upon KMT5 deletion, the colony growth of *F. graminearum* is somewhat inhibited, whereas the growth of *F. fujikuroi* remains unaffected (Bachleitner et al. 2021). Although KMT5 affects growth and virulence differently in these two species, it significantly affects the production of secondary metabolites (Bachleitner et al. 2021). In *Zymoseptoria tritici*, *KMT5* deletion mutants are more sensitive to genotoxic stress than the wild type (Moller et al. 2023). Moreover, the deletion of *KMT5* leads to significant differences in genome-wide transcription levels and has a large impact on chromatin modifications, including the complete loss of Ash1-mediated H3K36me3 and changes in the distribution of H3K27me3 and H3K4me2 in facultative heterochromatin regions (Moller et al. 2023).

## Crosstalk between different types of histone methylation

Among the types of histone methylation described above, interactions between different modifications are not limited to those within the same category (e.g., the competition for occupancy between the heterochromatin markers H3K9me and H3K27me). Indeed, interactions can also occur between different types of modifications (such as those that activate gene expression and those that suppress it), leading to the production of bivalent chromatin (Li et al. 2023). For instance, although activated and repressive modifications have mutually exclusive occupancy, altering their relative proportions can also regulate gene expression (Bernstein et al. 2006). Specifically, H3K4me and H3K27me often appear in different regions of the same gene, typically exerting opposite effects (Margueron and Reinberg 2011). H3K4me3 is primarily found at gene promoters, where it enhances gene expression, while H3K27me3 is located at gene silencing regions, where it inhibits gene expression. These opposing effects usually manifest as differences in the regulation of distinct regions of the same gene. In some cases, H3K4me and H3K27me modifications may also exhibit “competitive” characteristics (Bernstein et al. 2006). Sixteen bivalent-chromatin-regulated pathogenic genes (BCGs) have been identified in *F. graminearum*. Notably, the xylanase gene *BCG1* exhibits dynamic shifts in the ratio of H3K4me3 to H3K27me3 during infection of wheat (*Triticum aestivum*), which modulates its transcription pattern to facilitate host cell wall degradation and establish successful infection (Zhao et al. 2024). This “competitive modification” suggests that the balance between H3K4me and H3K27me plays an essential role in regulating gene activation or repression. Polycomb complexes (responsible for H3K27 methylation) and Trithorax complexes (responsible for H3K4 methylation) coordinate during specific developmental processes and may jointly regulate the on/off states of certain genes; their balance is crucial for the rapid fine-tuning of gene expression (Schuettengruber et al. 2017).

H3K4me and H3K9me are recognized and modulated by different protein complexes. H3K9me is maintained in a repressive state by HP1 (Jamieson et al. 2016), whereas H3K4me is preserved in an active transcriptional state through its corresponding modifying enzymes. During cellular differentiation or fate decisions, the interplay between H3K4me and H3K9me may play a key role in the activation and silencing of gene expression (Bilodeau et al. 2009). The relationship between H3K4me and the constitutive heterochromatin marker H3K9me is characterized by a competitive interaction, particularly in the same gene regulatory region (Bernstein et al. 2006). When H3K4 methylation increases, the level of H3K9 methylation generally decreases, and vice versa. This is because H3K4me typically marks transcriptionally active gene regions, whereas H3K9me is predominantly found in silenced regions, leading to spatial and functional exclusion between the two types of histone methylation (Du et al. 2021).

However, in some cases, H3K4me and H3K9me may act synergistically in different gene regions or cellular states to collectively maintain the epigenetic state of a gene. For example, H3K4me may enhance transcription at the promoter region of a gene, while H3K9me may help stabilize gene silencing in its regulatory regions. In other cases, the two modifications might also exhibit a synergistic effect. H3K4me and H3K9me do not directly compete but rather cooperate in different regions to regulate gene expression (Vermeulen et al. 2007). This additive effect allows for more refined and stable switching between transcriptional activity and silencing. In some cases, H3K4me and H3K9me exhibit alternating modification patterns across different chromatin regions. For example, in some eukaryotic genes, transcriptionally active regions are typically marked by H3K4me3, whereas transcriptionally silent regions are marked by H3K9me3 (Barski et al. 2007). This suggests that these two modifications exhibit spatiotemporal differences in their roles in regulating gene expression.

## Concluding remarks and perspectives

Epigenetic modifications, particularly histone methylation, play critical roles in regulating gene expression in filamentous fungi, thereby governing a wide range of biological processes, including mycelial growth, morphological differentiation, secondary metabolite biosynthesis, abiotic stress resistance, and pathogenicity. Whether in response to abiotic stress caused by environmental changes or during biological reactions to biotic stress, the precise regulation of gene expression is indispensable. Histone methylation regulates gene transcription by modifying chromatin structure, thereby affecting chromatin accessibility and modulating the binding of (and regulation by) transcription factors. Different types of histone methylation play key roles in these complex and diverse processes. For example, methylation of H3K4 and H3K36 is typically associated with transcriptional activation, while methylation of H3K27 and H3K9 is linked to transcriptional repression and genomic stability (Hyun et al. 2017).

Although extensive research on histone methylation has been conducted, studies on the crosstalk between different types of methylation and their catalytic activities remain limited, making these important topics for future investigations. Furthermore, while numerous studies have clarified the roles of histone methylation in directly regulating processes such as secondary metabolite biosynthesis (Chettri et al. 2018; Janevska et al. 2018a), appressorium development (Connolly et al. 2013; Hou et al. 2020), and virulence factor production in filamentous fungi (Lin et al. 2022; Wu et al. 2022), the accurate identification of target genes and the precise regulation of their expression still require further detailed exploration. The diverse types of histone methylation and their crosstalk in response to both biotic and abiotic stress represent important directions for future research.

During their interactions with their plant hosts, plant pathogens have developed a substantial degree of phenotypic plasticity in order to avoid and/or suppress recognition by the host. Such dynamic interactions drive the evolution of plant mechanisms that link pathogen sensing to rapid and effective defense activation to minimize fitness costs (Boller and Felix 2009; Huot et al. 2014). Most studies of plant–pathogen interactions have focused on chromatin dynamics employed by plants to adapt to and defend against pathogenic microbes. Limited information is available on how pathogenic microbes sense, adapt to, and invade their plant hosts, especially how they coordinate their chromatin dynamics.

Recent advancements in sequencing technologies have significantly broadened research approaches to histone modifications. Compared with traditional studies focusing on the impacts of histone modifications on gene expression, the emergence of single-cell sequencing and spatial omics now enables the systematic investigation of histone modification dynamics during fungal development, particularly their roles in regulating cell-type-specific differentiation. Furthermore, the integration of sequencing technologies with other biological methodologies has deepened our mechanistic understanding of histone modifications. For instance, flow cytometry coupled with sequencing allows the precise isolation of fungal cells at distinct stages of the cell cycle, providing a robust tool for elucidating how histone modifications are faithfully transmitted during mitosis. Moreover, the enrichment of fungal pathogens at distinct stages of infection via fluorescence-activated cell sorting offers a highly efficient strategy for profiling temporal changes in histone modifications during host invasion, thereby uncovering novel insights into the epigenetic regulation of pathogenic mechanisms in diverse fungal pathogens. These interdisciplinary approaches advance our ability to dissect the spatiotemporal complexity of the regulation of histone modifications during fungal development and host–pathogen interactions.

## Data Availability

This manuscript does not include any experimental data or materials or issues of ethics.
